# Editorial Note: Unmet need for family planning among reproductive-age women living with HIV in Ethiopia: A systematic review and meta-analysis

**DOI:** 10.1371/journal.pone.0327429

**Published:** 2025-07-09

**Authors:** The PLOS One Editors

This article [1] was identified as one of a series of articles for which the *PLOS One* Editors have concerns about authorship and adherence to the journal’s first and second publication criteria. We regret that the issues were not addressed prior to the article’s publication.

## References

[1] Kefale B, Adane B, Damtie Y, Arefaynie M, Yalew M, Andargie A, et al. Unmet need for family planning among reproductive-age women living with HIV in Ethiopia: A systematic review and meta-analysis. PLoS ONE. 2021;16(8):e0255566. doi: 10.1371/journal.pone.0255566 34339464 PMC8328287

